# Price changes of antibiotics and perceived substitutes in the context of OTC policy changes in Mexico and Brazil

**DOI:** 10.1186/2052-3211-8-S1-O11

**Published:** 2015-10-05

**Authors:** Yared Santa-Ana-Tellez, Veronika J Wirtz, Hubert GM Leufkens, Aukje K Mantel-Teeuwisse

**Affiliations:** 1 WHO Collaborating Centre for Pharmaceutical Policy & Regulation, Utrecht Institute for Pharmaceutical Sciences (UIPS), Utrecht, 3584 CG, the Netherlands; 2 Department of Global Health, Boston University School of Public Health, Boston, MA 02118, USA; 3 Center for Health Systems Research, National Institute of Public Health, Cuernavaca, C.P. 62100, Mexico

## Problem statement

The over-the-counter (OTC) sales restriction of antibiotics in Mexico and Brazil might have created incentives to maintain the sales revenue by increasing antibiotics prices and attract the consumption of substitutes by decreasing their price.

## Objectives

To explore price changes of antibiotics and their substitutes in Mexico and Brazil.

Policies targeted: Over-the-counter sales restriction of antibiotics in Mexico and Brazil.

Stakeholders: Governments of Mexico and Brazil.

Region covered: PAHO region. Private sector at national level.

## Methods

Study design: Quasi-experimental.

Time period: January 2008-March 2013.

Setting: IMS Health retail quarterly trade prices from the private sectors in Mexico and Brazil. Groups studied were: antibiotics, cough-and-cold medicines, non-steroidal anti-inflammatory drugs (NSAIDs), and analgesics. The latter two groups were combined in the analysis of median price per defined daily dose (DDD). All prices were adjusted for inflation rate and converted to US Dollars.

Interventions: Interrupted time series analysis to measure price changes (level and slope) after the OTC sales regulation of antibiotics and seasonal price changes. Stationarity and autocorrelation corrected using ARIMA models.

## Results

After the regulation in Mexico the median price of antibiotics increased by $0.6 per DDD (p<0.001, level increase) with a slope decrease of $0.21 per quarter (p<0.001), NSAIDs-analgesics prices decreased by $0.11 (p=0.04) with a slope increase of $0.02 (p=0.002) per quarter, and prices of cough-and-cold medicines increased by $0.36 (p=0.006). In Brazil prices of antibiotics did not change after the regulation, NSAIDs-analgesics median price increased by $0.04 (p<0.00) with a slope decrease of $0.01 (p=0.001) per quarter, and cough-and-cold medicines price slope increased by $0.02 (p<0.00) per quarter. For both countries we observed seasonal variation in prices with highest prices during warm seasons where use is relatively low (spring and summer). The difference in price of antibiotics between winter and summer was $1.5 (p<0.001) in Mexico and $0.31 (p=0.02) in Brazil, and for cough-and-cold medicines this difference was $3.07 (p<0.001) in Mexico and $0.58 (p<0.001) in Brazil. The difference in price of NSAIDs-analgesics between winter and autumn in Mexico was $0.17 (p<0.001) and in Brazil was $0.09 (p<0.001).

## Conclusions

After the regulation in Mexico prices of antibiotics increased while prices of NSAIDs-analgesics decreased. In Brazil NSAIDs-analgesics prices increased. However these changes were outweighed by the seasonal variation in prices.

## Lessons learned and success factors

Possible effects on prices need to be considered when designing and implementing pharmaceutical polices, to anticipate or prevent unintended outcomes.

